# Deferred and referred deliveries contribute to stillbirths in the Indian state of Bihar: results from a population-based survey of all births

**DOI:** 10.1186/s12916-019-1265-1

**Published:** 2019-02-07

**Authors:** Rakhi Dandona, G. Anil Kumar, Md. Akbar, Debarshi Bhattacharya, Priya Nanda, Lalit Dandona

**Affiliations:** 1 Public Health Foundation of India, Sector 44, Institutional Area, Gurugram, National Capital Region India; 2 Institute for Health Metrics and Evaluation, University of Washington, Seattle, USA; 3 Bill & Melinda Gates Foundation, India Country Office, New Delhi, India

**Keywords:** Antepartum, Deferred delivery, India, Intrapartum, Rate, Stillbirth

## Abstract

**Background:**

The India Newborn Action Plan (INAP) aims for < 10 stillbirths per 1000 births by 2030. A population-based understanding of risk factors for stillbirths compared with live births that could assist with reduction of stillbirths is not readily available for the Indian population.

**Methods:**

Detailed interviews were conducted in a representative sample of all births between January and December 2016 from 182,486 households (96.2% participation) in 1657 clusters in the Indian state of Bihar. A stillbirth was defined as foetal death with gestation period of ≥ 7 months wherein the foetus did not show any sign of life. The association of stillbirth was investigated with a variety of risk factors among all births using a hierarchical logistic regression model approach.

**Results:**

A total of 23,940 births including 338 stillbirths were identified giving the state stillbirth rate (SBR) of 15.4 (95% CI 13.2–17.9) per 1000 births, with no difference in SBR by sex. Antepartum and intrapartum SBR was 5.6 (95% CI 4.3–7.2) and 4.5 (95% CI 3.3–6.1) per 1000 births, respectively. Detailed interview was available for 20,152 (84.2% participation) births including 275 stillbirths (81.4% participation). In the final regression model, significantly higher odds of stillbirth were documented for deliveries with gestation period of ≤ 8 months (OR 11.36, 95% CI 8.13–15.88), for first born (OR 5.79, 95% CI 4.06–8.26), deferred deliveries wherein a woman was sent back home and asked to come later for delivery by a health provider (OR 5.51, 95% CI 2.81–10.78), and in those with forceful push/pull during the delivery by the health provider (OR 4.85, 95% CI 3.39–6.95). The other significant risk factors were maternal age ≥ 30 years (OR 3.20, 95% CI 1.52–6.74), pregnancies with multiple foetuses (OR 2.82, 95% CI 1.49–5.33), breech presentation of the baby (OR 2.70, 95% CI 1.75–4.18), and births in private facilities (OR 1.75, 95% CI 1.19–2.56) and home (OR 2.60, 95% CI 1.87–3.62). Varied risk factors were associated with antepartum and intrapartum stillbirths. Birth weight was available only for 40 (14.5%) stillborns. Among the facility deliveries, the women who were referred from one facility to another for delivery had significantly high odds of stillbirth (OR 3.32, 95% CI 2.03–5.43).

**Conclusions:**

We found an increased risk of stillbirths in deferred and referred deliveries in addition to demographic and clinical risk factors for antepartum and intrapartum stillbirths, highlighting aspects of health care that need attention in addition to improving skills of health providers to reduce stillbirths. The INAP could utilise these findings to further strengthen its approach to meet the stillbirth reduction target by 2030.

**Electronic supplementary material:**

The online version of this article (10.1186/s12916-019-1265-1) contains supplementary material, which is available to authorized users.

## Background

The Indian government adopted a target of < 10 stillbirths per 1000 births by 2030, the first ever national stillbirth prevention target, in 2014 as part of the India Newborn Action Plan (INAP) [1]. This is an important step in the recognition of stillbirth as a public health concern as India has the largest burden of stillbirths globally with an estimated 590,000 stillbirths in 2015 [2].

We have previously reported a stillbirth rate (SBR) of 21.2 (95% CI 19.7–22.6) per 1000 births for the Indian state of Bihar in 2012–2013, which were the first state-wide robust population-based estimates of stillbirth from India given the significant underreporting of stillbirths in the Sample Registration System and the Demographic Health Surveys [1, 3, 4]. We reported on the risk factors for stillbirths and also highlighted the missed opportunities in the health system to reduce stillbirths [3]. However, as no comparative data were available on livebirths in the previous survey, hence, the acute need remained to better understand risk factors for stillbirths among all births that could further assist with reduction of stillbirths [3]. We now address this gap by reporting detailed epidemiology of stillbirths from a population-based survey of all births wherein the risk factors were documented for each birth irrespective of the birth outcome from Bihar state. This Indian state, with a population of 104 million in year 2016, is the third most populous state, with 11% of it being urban [5].

## Methods

### Survey design

The Bill & Melinda Gates Foundation supports the Bihar Technical Support Programme, with the long-term goals of supporting the Government of Bihar in reducing maternal, newborn, and child mortality; improving family planning services; and reducing under nutrition rates in the state [6]. A neonatal mortality rate of 32.2 (95% CI 27.6–36.8) was estimated in 2011 for Bihar in the baseline survey at the start of this programme [7]. The current survey was designed with a sample size of 23,200 births to detect a change of 17.5% in neonatal mortality rate in Bihar from 2011 to 2016 with 80% power. The survey aimed to obtain a representative sample of women with a birth between January and December 2016 in Bihar. The stillbirth assessment was carried out as part of this survey.

For the survey, the women were selected by a multistage sampling procedure from Bihar state which is divided into 38 districts, each of which is divided into 5–27 blocks, giving a total of 534 blocks in the state. To obtain a representative sample of women with a birth between January and December 2016 from 50% of the 534 blocks for the study, we first stratified the 534 blocks as with only rural population (70.2%) and those with both rural and urban populations (29.8%). We sampled 267 blocks which included 187 (70%) blocks with only rural population and 80 (30%) blocks with both rural and urban populations. Within these 267 blocks, the secondary sampling units (SSUs) were villages in rural areas and urban frame survey blocks in urban areas as defined by the Census 2011 [8]. The SSUs with < 75 households were combined with an adjacent SSU, and the large rural SSUs were split into equal sized segments of 75–100 households using natural boundaries. A total of 1657 SSUs (1475 rural and 182 urban) were sampled in proportion to the number of SSUs in each block, using simple random systematic sampling without replacement across all the 267 sampled blocks.

### Data collection

Each selected SSU was mapped, and all the households (a household was defined as people eating from the same kitchen) enumerated. During the enumeration, trained interviewers documented the birth outcomes between January and December 2016 among women aged 15–49 years in each household. Date of birth, sex of the baby born, and whether it was a livebirth or stillbirth were documented for each birth. Stillbirths were documented in enumeration by confirming that the baby did not show any sign of life (did not cry, breathe, and move) in order to differentiate stillbirths and neonatal deaths soon after delivery. We also documented births between January and December 2016 for women who had died during or after giving birth to ensure a robust estimation of total births in this population.

Following enumeration, all women who had reported a birth irrespective of the outcome were eligible for a detailed interview. During the interview, after documenting the background information including socio-demographic characteristics of the participants, questions were asked again to differentiate a stillbirth (did not cry, breathe, and move) from a neonatal death that occurred soon after delivery. We documented maternal birth history and the pregnancy, labour, and delivery details for all births. For stillbirths, photographs of macerated and fresh stillborn babies were shown to the respondents to document baby’s appearance at birth, and when the baby’s last movements were felt by the mother was documented.

Data for the study were collected between March and October 2017. The questionnaire was developed in English and then translated into Hindi (local language), after which these were back-translated into English to ensure the accurate and relevant meaning and intent of the questions. Pilot testing of the questionnaire was carried out and modifications made as necessary. Interviews were captured using the Open Development Kit software in hand-held tablets. Data entered were scrutinised using the internal consistency checks built in to detect and correct errors using the procedures standardised in the baseline study to meet the data quality. About 30% of the data were collected by the interviewers under direct supervision, and an additional 5% of the interviews were checked by the supervisors by visiting the respondent again.

### Analysis

A stillbirth was defined as a foetal death with gestation period of ≥ 7 months wherein the baby did not show any sign of life (did not cry, breathe, and move). Before the analysis, data from all stillbirth interviews were reviewed to re-confirm that a stillbirth was reported based on the signs of life and gestational age. A total of ten cases reported as stillbirth were considered as miscarriage as the gestation period at the time of birth was mentioned as ≤ 6 months. In addition, we reviewed the interviews of neonates who had died on day 0 of birth to check for possible misreporting between neonatal death and stillbirth. Three neonatal deaths were reassigned as stillbirths, and no stillbirth was reassigned as a neonatal death. Data on gestation period was reported in months by the participants as is the practice in India; we assumed 1 month as 4 weeks to identify stillbirths for this analysis.

We estimated the overall stillbirth rate (SBR) and SBR in boys and girls for Bihar state in the year 2016. The sampling weight with design effect was applied to the rates to adjust for Bihar’s population, and 95% confidence interval (CI) is reported. The change in SBR from 2012 to 2016 for Bihar was also estimated [3]. We classified the stillbirths as antepartum and intrapartum (fresh stillbirth) [9], based on the baby’s last movement felt by the mother as an indication of the time of death and the description of stillborn baby (fresh or macerated), with preference given to the baby’s movement over description as described in detail elsewhere [3, 10–14]. We investigated the association of stillbirth, antepartum stillbirth, and intrapartum stillbirth with a variety of potential risk factors including socio-demographic factors, maternal risk factors, and pregnancy-, labour-, and delivery-related factors among all births. Based on our previous assessment of the health provider interface for stillbirths [3], we included “deferred delivery” as a variable in these models to indicate if a woman had reported to a health provider for delivery but she was sent back by the health provider to come later for delivery. Distribution and results of unadjusted simple logistic regression are reported for all potential risk factors that were assessed. Furthermore, we explored the associations between potential risk factors for stillbirth, antepartum stillbirth, and intrapartum stillbirth using a hierarchical approach to build the logistic regression model that gave importance to distal determinants of stillbirths [15, 16]. We ran five models with each model adjusted for place of residence and sex of the baby, and each sequential model incorporated variables from the preceding model if *p* was < 0.2 (value for at least one category to be < 0.2 for multiple category variables) [17]. Birthweight was not considered in the adjusted logistic regression as it was not available for 84.6% of the stillbirth cases. Odds ratio with 95% CI is presented for all models of regression results.

Following from our previous assessment of the health provider interface for stillbirths [3], in the current survey for the women who had delivered in a health facility, we documented if they had gone to another facility for delivery prior to delivering where they did deliver (termed as “referral”). We present the referral pattern, reasons for referral, and risk of stillbirths in such cases as compared with those who were not referred. All analysis was performed using STATA 13.1 software (Stata Corp, USA).

## Results

A total of 23,940 births in year 2016 were identified during the enumeration from 182,486 households (96.2% participation) covering a population of 945,216. Three hundred and thirty-eight stillbirths were identified giving an estimated SBR of 15.4 (95% CI 13.2–17.9) per 1000 births for the state. The SBR for boys was 15.7 (95% CI 12.7–19.2) and for girls was 14.6 (95% CI 11.5–18.5) per 1000 births.

Of the total births identified in enumeration, detailed interview was available for 20,152 (84.2% participation) births including 275 stillbirths (81.4% participation). Table 1 documents the distribution of all births and stillbirths, and results of unadjusted logistic regression for the risk factors assessed for stillbirths. A few of the socio-demographic, maternal, and pregnancy-related risk factors and nearly all of the risk factors during the labour and delivery periods had a significantly higher unadjusted risk of stillbirth (Table 1). Similar results were seen for risk factors for stillbirth during labour and delivery by the place of delivery (Additional file 1).

**Table 1:** Basic descriptive and results of unadjusted logistic regression for socio-demographic, maternal, pregnancy, labour, and delivery risk factors for all births including stillbirths between January and December 2016 in the Indian state of Bihar. OR denotes odds ratio and CI denotes confidence interval

Risk factor		All births *N* = 20,152 (%)	Stillbirths *N* = 275 (%)	Unadjusted OR (95% CI)
Socio-demographics				
Place of residence	Rural	18,388 (91.3%)	262 (95.3%)	1.95 (1.11–3.41)
	Urban	1764 (8.8%)	13 (4.7%)	1.00
Wealth index quartile^1^	I	5013 (24.9%)	75 (27.5%)	1.22 (0.87–1.71)
	II	5003 (24.9%)	68 (24.9%)	1.11 (0.78–1.57)
	III	5038 (25.1%)	68 (24.9%)	1.10 (0.78–1.56)
	IV	5048 (25.1%)	62 (22.7%)	1.00
Type of cooking fuel used^2^	Solid	7033 (34.9%)	91 (33.2%)	1.00
	Non-solid	13,108 (65.1%)	183 (66.8%)	1.08 (0.84–1.39)
Maternal				
Maternal age^3^	15–19 years	939 (4.7%)	15 (5.5%)	1.00
	20–24 years	8418 (41.8%)	131 (47.6%)	0.97 (0.57–1.67)
	25–29 years	7656 (38.0%)	86 (31.3%)	0.70 (0.40–1.22)
	≥ 30 years	3136 (15.6%)	43 (15.6%)	0.86 (0.47–1.55)
Any tobacco use ever	Yes	527 (2.6%)	9 (3.3%)	1.26 (0.65–2.47)
	No	19,625 (97.4%)	266 (96.7%)	1.00
First born	Yes	4876 (24.2%)	143 (52.0%)	3.47 (2.73–4.40)
	No	15,276 (75.8%)	132 (48.0%)	1.00
Previous history of stillbirth	Yes	980 (4.9%)	19 (6.9%)	1.46 (0.91–2.34)
	No	19,172 (95.1%)	256 (93.1%)	1.00
Ever miscarriage^4^	Yes	2753 (13.7%)	41 (15.0%)	1.11 (0.80–1.56)
	No	17,389 (86.3%)	233 (85.0%)	1.00
Diabetes mellitus irrespective of pregnancy ^5^	Yes	66 (0.3%)	4 (1.5%)	4.79 (1.73–13.26)
	No	19,714 (99.7%)	262 (98.5%)	1.00
Hypertension irrespective of pregnancy^6^	Yes	344 (1.7%)	9 (3.4%)	2.02 (1.03–3.95)
	No	19,465 (98.3%)	256 (96.6%)	1.00
Pregnancy				
At least one antenatal care visit during pregnancy^7^	Yes	16,363 (81.2%)	212 (78.2%)	1.00
	No	3784 (18.8%)	59 (21.8%)	1.21 (0.90–1.61)
Received 2 tetanus toxoid injections during pregnancy^8^	Yes	16,295 (81.1%)	204 (74.2%)	1.00
	No	3806 (18.9%)	71 (25.8%)	1.50 (1.14–1.97)
Consumed iron folic acid tablets during pregnancy^9^	Yes	8091 (40.4%)	84 (30.8%)	1.00
	No	11,941 (59.6%)	189 (69.2%)	1.53 (1.18–1.99)
Pregnancy with multiple foetuses	Yes	354 (1.8%)	18 (6.6%)	4.07 (2.50–6.65)
	No	19,798 (98.2%)	257 (93.5%)	1.00
Hypertension in the last trimester of pregnancy^10^	Yes	515 (2.6%)	11 (4.1%)	1.63 (0.89–3.01)
	No	19,353 (97.4%)	255 (95.9%)	1.00
Malaria in the last trimester of pregnancy^11^	Yes	455 (2.3%)	6 (2.2%)	0.96 (0.43–2.18)
	No	19,328 (97.7%)	264 (97.8%)	1.00
Syphilis during pregnancy^12^	Yes	84 (0.4%)	1 (0.4%)	0.88 (0.12–6.33)
	Do not know	1169 (5.8%)	18 (6.6%)	1.14 (0.70–1.84)
	No	18,898 (93.8%)	256 (93.1%)	1.00
Fever in the last 3 months of pregnancy^13^	Yes	3596 (18.0%)	49 (18.0%)	1.00 (0.73–1.36)
	No	16,353 (82.0%)	223 (82.0%)	1.00
Convulsions in the last 3 months of pregnancy^14^	Yes	2245 (11.3%)	30 (11.1%)	0.98 (0.67–1.43)
	No	17,573 (88.7%)	240 (88.9%)	1.00
Mother was informed that the baby was not growing adequately inside the womb^15^	Yes	703 (3.5%)	24 (8.8%)	2.68 (1.75–4.10)
	No	19,209 (96.5%)	250 (91.2%)	1.00
Gestation period	7 months	179 (0.9%)	39 (14.2%)	27.7 (18.9–40.6)
	> 7–8 months	378 (1.9%)	41 (14.9%)	12.1 (8.5–17.2)
	> 8 months	19,595 (97.2%)	195 (70.9%)	1.00
Labour				
Mother had come for delivery earlier but was asked to come later for delivery (deferred delivery)^13^	Yes	175 (0.9%)	15 (5.5%)	7.06 (4.10–12.16)
	No	19,774 (99.1%)	259 (94.5%)	1.00
Spontaneous labour^16^	Yes	14,801 (74.0%)	164 (63.6%)	1.00
	No	5214 (26.1%)	94 (36.4%)	1.64 (1.27–2.12)
Foul smelling discharge^17^	Yes	1005 (5.0%)	29 (10.6%)	2.27 (1.53–3.35)
	No	19,014 (95.0%)	246 (89.5)	1.00
Delivery				
Place of delivery^18^	Public facility	10,712 (53.2%)	89 (33.0%)	1.00
	Private facility	3394 (16.9%)	80 (29.6%)	2.88 (2.13–3.91)
	Home	6023 (29.9%)	101 (37.4%)	2.04 (1.53–2.71)
Mother had gone to a health facility for delivery but delivered in another facility (referred delivery)^19^	Yes	827 (5.9%)	46 (27.5%)	6.40 (4.53–9.06)
	No	13,275 (94.1%)	121 (72.5%)	1.00
Vaginal delivery^20^	Yes	18,096 (89.9%)	229 (86.1%)	0.69 (0.49–0.98)
	No	2028 (10.1%)	37 (13.9%)	1.00
Push/forceful pull done during delivery by the health provider^21^	Yes	1025 (5.2%)	60 (22.8%)	5.68 (4.23–7.63)
	No	18,736 (94.8%)	203 (77.2%)	1.00
Entangled cord around the baby’s neck^22^	Yes	782 (3.9%)	21 (7.6%)	2.03 (1.29–3.19)
	Do not know	1766 (8.8%)	18 (6.6%)	0.76 (0.47–1.23)
	No	17,587 (87.4%)	236 (85.8%)	1.00
Breech position of the baby^23^	Yes	674 (3.4%)	41 (15.5%)	5.49 (3.90–7.73)
	No	19,111 (96.6%)	223 (84.5%)	1.00
Birthweight of the baby (kilogrammes)^24^	≥2.0	12,896 (64.7%)	37 (14.3%)	1.00
	< 2.0	582 (2.9%)	3 (1.2%)	1.80 (0.55–5.86)
	Not weighed	4704 (23.6%)	0 (0.0%)	(empty)
	Do not know if weighed	1766 (8.9%)	219 (84.6%)	49.20 (34.59–69.98)

Data was not available for ^1^50 (0.25%) births, ^2^11 (0.05%), ^3^3 (0.01%), ^4^10 (0.05%), ^5^372(1.85%), ^6^343 (1.70%), ^7^5 (0.02%), ^8^51 (0.25%), ^9^120 (0.60%), ^10^284 (1.41%), ^11^369 (1.83%), ^12^1 (0.005%), ^13^203 (1.01%), ^14^334 (1.66%), ^15^240 (1.19%), ^16^137 (0.68%), ^17^133 (0.66%), ^18^23 (0.11%), ^19^4 (0.03%) data shown only for women who delivered in a health facility, ^20^28 (0.14%), ^21^391 (1.94%), ^22^17 (0.08%), ^23^367 (1.82%), and ^24^204 (1.01%) birth

A significantly higher proportion of births was stillborn (8.6%, *p* < 0.001) among women (175, 0.9%) for whom the delivery was deferred (Table 1). Of these women, 143 (81.7%) mentioned that they were sent back home as the health provider informed them that “there was still time for delivery”. The proportion of deferred deliveries (Additional file 1) was significantly higher in the private facilities (2.1%, *p* < 0.001) as compared with public facilities or home deliveries, and in breech position (2.8%) as compared with normal position deliveries (0.8%, *p* < 0.001).

Birth weight measurement was reported only for 40 (14.5%) of stillbirths (Table 1), and women did not know if birth weight was taken in 219 (84.6%) stillbirths. Considering the size of baby at birth, 42.2% the stillborns were reported to be of an average size, 37.5% as small/very small, and 10.2% as larger than usual in size. For the 703 (3.5%) births where the mother was informed during pregnancy that baby was not growing adequately (Table 1), 41 (5.8%) were < 2.0 kg, 474 (67.4%) were ≥ 2.0 kg and 105 (14.9%) were not weighed at birth.

After adjustment for the place of residence and sex of the baby in the final sequential logistic regression model (Table 2), gestation period of ≤ 8 months (OR 11.36, 95% CI 8.13–15.88) had the highest odds for stillbirth followed by first born births (OR 5.79, 95% CI 4.06–8.26), deferred deliveries (OR 5.51, 95% CI 2.81–10.78), and those reporting push/pull (manual fundal pressure/forceful pulling of the baby) during the delivery by health provider (OR 4.85, 95% CI 3.39–6.95). In addition, maternal age of ≥ 30 years, pregnancies with multiple foetuses, and all the labour and delivery period risk factors except foul smelling discharge had a significantly higher odds of stillbirth (Table 2). Births in private facilities and home (OR 2.23, 95% CI 1.67–2.99) had a significantly higher risk of stillbirth. As the deferred deliveries had nearly sixfold higher odds of a stillbirth (Table 2), these were explored further by place of delivery to understand the role of breech position of the baby, and push/forceful pull performed during delivery by the health provider. Significantly higher odds of stillbirth were seen with deferred deliveries that were delivered at home, with breech position of the baby, and if push/forceful pull during delivery was performed by the health provider (Additional file 2).

**Table 2:** Results of sequential multiple logistic regression models for association of stillbirths with socio-demographic, maternal, pregnancy, labour, and delivery-related risk factors in the Indian state of Bihar. Statistically significant odds ratios are shown in italics in the final model 5

Risk factor	Adjusted odds ratio for stillbirth (95% confidence interval)				
	Model 1*	Model 2*	Model 3*	Model 4*	Model 5*
Rural place of residence	1.89 (1.07–3.34)	1.92 (1.08–3.39)	1.88 (1.05–3.35)	2.02 (1.12–3.62)	1.71 (0.92–3.16)
Boy baby	1.21 (0.95–1.53)	1.29 (1.01–1.65)	1.42 (1.09–1.84)	1.36 (1.04–1.78)	1.25 (0.95–1.65)
Wealth index quartile					
I	1.12 (0.80–1.58)^‡^				
II	1.02 (0.72–1.45)^‡^				
III	1.03 (0.72–1.46)^‡^				
IV	1.00				
Maternal age					
15–19 years		1.00	1.00	1.00	1.00
20–24 years		1.57 (0.89–2.75)	1.59 (0.87–2.88)	1.59 (0.88–2.90)	1.73 (0.93–3.20)
25–29 years		2.28 (1.24–4.20)	2.27 (1.19–4.33)	2.27 (1.18–4.34)	*2.63 (1.35–5.14)*
≥ 30 years		3.13 (1.59–6.14)	3.18 (1.57–6.46)	3.34 (1.64–6.80)	*3.20 (1.52–6.74)*
Solid cooking fuel use		1.06 (0.82–1.38)^‡^			
Any tobacco use ever		1.45 (0.73–2.86)^‡^			
First born		5.15 (3.78–7.01)	5.34 (3.82–7.47)	5.38 (3.83–7.56)	*5.79 (4.06–8.26)*
Previous history of stillbirth		1.41 (0.84–2.37)	1.46 (0.85–2.50)	1.25 (0.72–2.18)^‡^	
Previous history of miscarriage		1.13 (0.80–1.60)^‡^			
Maternal history of diabetes mellitus irrespective of pregnancy		2.84 (0.89–9.08)	3.25 (1.00–10.53)	3.04 (0.97–9.56)	1.84 (0.55–6.15)
Maternal history of high blood pressure irrespective of pregnancy		1.43 (0.64–3.20)^‡^			
No maternal antenatal care visit during pregnancy			1.42 (1.02–1.99)	1.55 (1.11–2.16)	1.32 (0.93–1.88)
Mother did not receive 2 tetanus toxoid injections during pregnancy			1.53 (1.11–2.12)	1.48 (1.06–2.05)	1.35 (0.95–1.90)
Mother did not consume iron folic acid tablets during pregnancy			1.23 (0.93–1.63)	1.15 (0.87–1.53)^‡^	
Pregnancy with multiple foetuses			2.80 (1.52–5.18)	3.33 (1.84–6.04)	*2.82 (1.49–5.33)*
Maternal hypertension in the last trimester of pregnancy			1.32 (0.65–2.69)^‡^		
Mother had malaria in the last trimester of pregnancy			0.83 (0.34–2.02)^‡^		
Mother diagnosed with syphilis during this pregnancy					
No			1.00		
Yes			1.74 (0.23–13.00)^‡^		
Do not know			1.04 (0.61–1.77) ^‡^		
Mother had fever in the last 3 months of pregnancy			1.05 (0.74–1.49)^‡^		
Mother had convulsions in the last 3 months of pregnancy			0.86 (0.56–1.32)^‡^		
Mother was informed that the baby was not growing adequately inside the womb			1.75 (1.04–2.94)	1.34 (0.80–2.25) ^‡^	
Gestation period					
7 months			21.63 (14.16–33.03)	21.57 (13.93–33.39)	*19.92 (12.41–31.97)*
> 7–8 months			9.97 (6.74–14.74)	9.06 (6.05–13.58)	*8.41 (5.48–12.92)*
> 8 months			1.00	1.00	1.00
Deferred delivery				5.31 (2.81–10.04)	*5.51 (2.81–10.78)*
Spontaneous labour				1.57 (1.19–2.08)	*1.55 (1.15–2.09)*
Foul smelling discharge				1.59 (1.00–2.53)	1.38 (0.84–2.26)
Place of delivery					
Public facility					1.00
Private facility					*1.75 (1.19–2.56)*
Home					*2.60 (1.87–3.62)*
Vaginal delivery					*1.76 (1.08–2.88)*
Push/forceful pull done during delivery by the health provider					*4.85 (3.39–6.95)*
Entangled cord around the baby’s neck					
No					1.00
Yes					1.41 (0.83–2.37)
Do not know					*0.34 (0.16–0.72)*
Breech presentation of the baby					*2.70 (1.75–4.18)*

*Model adjusted for sex of the baby and place of residence

^‡^*p* ≥ 0.2, hence excluded from the sequential model

**Fig. 1:**
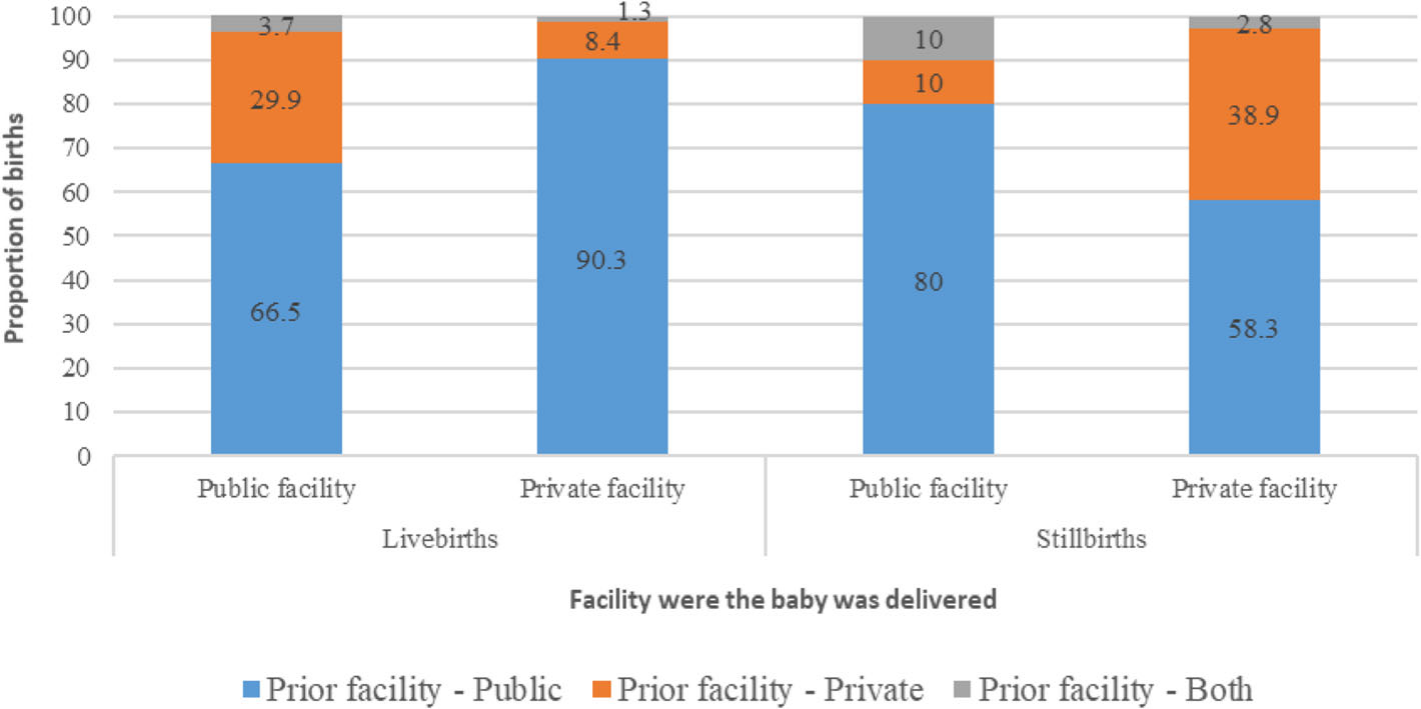
Referral pattern for 827 births where the women had gone to another facility for delivery but then delivered in a different facility in the Indian state of Bihar. Data missing on 9 births

A total of 827 (5.9%) among the 14,106 women who had a facility delivery were considered as “referred” (Table 1). These referral births were significantly more likely to be stillborn (5.6%, *p* < 0.001) as compared with non-referred births (0.9%). Considering these referral births, the prior facility was public facility for the majority (687, 83.1%) followed by private facility (115, 13.9%), 16 (1.9%) had gone to both types of facilities, and 9 (1.1%) elsewhere. A varied referral pattern was seen for livebirths and stillbirths (Fig. 1). A significantly (*p* < 0.001) higher proportion of referrals between private facilities was seen for stillbirths (38.9%) than for livebirths (8.4%); however, a significantly higher proportion of referrals from private to public facility was seen for livebirths (29.9%) than for stillbirths (10%). The major reasons for moving between facilities for delivery were (not mutually exclusive) the health provider asked to go to another facility (64.6%), the health provider refused treatment (23.3%), long wait at the prior facility (14%), and the woman did not like services at the prior facility (11.6%). These reasons were similar for livebirths and stillbirths except that among those with stillbirths, 8.7% mentioned “the health provider had refused to deliver dead baby” as the reason to move to another facility. Considering all births (Additional file 3), nurses delivered 45.1%, untrained Dai 20.1%, and doctors 15.9% of the babies, with the proportion of stillbirths significantly higher in births delivered by others (*p* < 0.001; untrained and unskilled people). Among the facility births (Additional file 3), the proportion of deliveries by doctors was higher in the referred than non-referred births (55.5% vs 19.9%) with proportion of stillbirths significantly higher in the former (6.54%; *p* < 0.001). Similar results were seen for deliveries by a nurse at a facility based on referral (*p* < 0.001; Additional file 3). When considering only facility deliveries and inclusion of referral deliveries as a risk factor in the sequential logistic regression model (Additional file 4), the referred deliveries were 3.32 times more likely to result in a stillbirth. The referred deliveries were explored further to understand the role of breech position of the baby, and push/ forceful pull performed during delivery by the health provider by place of delivery. Significantly high odds of stillbirth were seen with referred deliveries that were at public facilities and with breech position of the baby (Additional file 5).

**Fig. 2:**
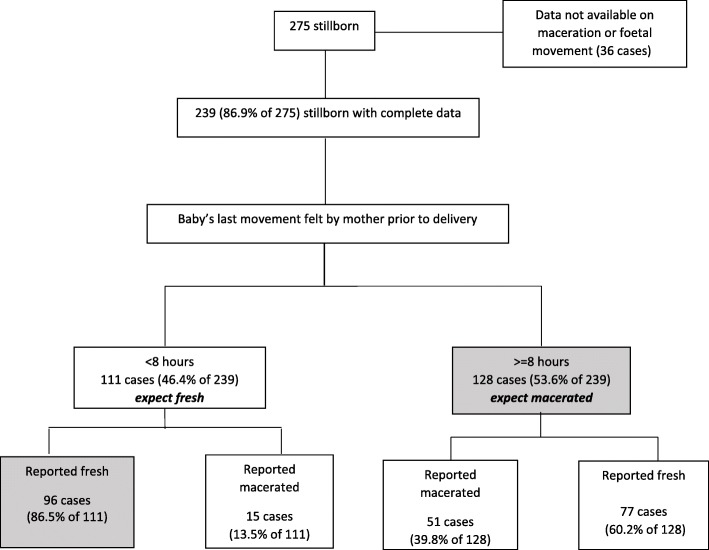
Classification process used to identify antepartum and intrapartum deaths for the stillbirths in the Indian state of Bihar

Figure 2 summarises the classification process for the identification of antepartum and intrapartum deaths. For the 239 (86.9%) stillborn for whom data both on the baby’s movement felt by mother before delivery and skin maceration were available, 128 (53.6%) were considered as antepartum (5.6, 95% CI 4.3–7.2 antepartum stillbirths per 1000 births), 96 (40.2%) as intrapartum deaths (4.5, 95% CI 3.3–6.1 intrapartum stillbirths per 1000 births, and the rest (6.2%) could not be classified. The reported mismatch between the baby’s movement and appearance was more pronounced for antepartum deaths wherein fresh appearance was reported for 60.2% of the cases (Fig. 2). The distribution of stillbirths with gestation period of 7 months was 14.1% and 6.2%, with 8 months was 21.9% and 8.3%, and with > 8 months was 64.1% and 85.4% among antepartum and intrapartum stillbirths, respectively. Boys accounted for 56.3%, 55.2%, and 58.8% of antepartum, intrapartum, and non-classifiable stillbirths, respectively (*p* = 0.915).

The risk factors associated with antepartum and intrapartum stillbirths based on the sequential logistic regression model differed (Table 3). After adjustment for place of residence and sex of the baby in the final sequential logistic regression model, gestation period of ≤ 8 months was significantly associated with both types of stillbirth though the odds for antepartum (OR 16.09, 95% CI 10.39–24.91) were 3.5 times more than that for intrapartum stillbirths (OR 4.53, 95% CI 2.40–8.55). Deferred delivery (OR 5.61, 95% CI 2.23–14.13), no antenatal care during pregnancy (OR 1.99, 95% CI 1.25–3.16), foul smelling discharge (OR 2.04, 95% CI 1.10–3.78), and private facility birth (OR 2.00, 95% CI 1.15–3.46) had significantly higher odds of antepartum but not intrapartum stillbirth. On the other hand, breech position of the baby (OR 4.33, 95% CI 2.38–7.89) and women from households using solid fuel (OR 1.70, 95% CI 1.01–2.88) had significantly higher odds of intrapartum but not antepartum stillbirth. Higher maternal age, first born, those who delivered at home, and those reporting push/forceful pull during the delivery by health provider were significantly associated with both types of stillbirth.

**Table 3:** Results of sequential multiple logistic regression models for association of antepartum and intrapartum stillbirths with socio-demographic, maternal, pregnancy, labour, and delivery-related risk factors in the Indian state of Bihar. Statistically significant odds ratios are shown in italics in the final model 5

Risk factor	Adjusted odds ratio for stillbirth (95% confidence interval)				
	Model 1*	Model 2*	Model 3*	Model 4*	Model 5*
Antepartum stillbirth					
Rural place of residence	3.16 (1.15–8.68)	3.08 (1.12–8.44)	2.81 (1.01–7.77)	3.02 (1.09–8.36)	2.98 (0.92–9.60)
Boy baby	1.18 (0.83–1.67)	1.31 (0.90–1.88)	1.31 (0.90–1.92)	1.19 (0.82–1.74)	1.09 (0.73–1.61)
Wealth index quartile					
I	0.81 (0.49–1.33)^‡^				
II	0.83 (0.51–1.37)^‡^				
III	0.87 (0.53–1.43)^‡^				
IV	1.00				
Maternal age					
15–19 years		1.00	1.00	1.00	1.00
20–24 years		1.99 (0.79–4.99)	1.92 (0.75–4.91)	2.51 (0.89–7.12)	2.60 (0.90–7.50)
25–29 years		2.90 (1.08–7.79)	2.57 (0.94–7.01)	3.28 (1.09–9.91)	*3.37 (1.09–10.41)*
≥ 30 years		4.36 (1.50–12.65)	3.86 (1.31–11.34)	5.09 (1.58–16.34)	*4.63 (1.39–15.44)*
Solid cooking fuel use		0.86 (0.59–1.25)^‡^			
Any tobacco use ever		2.64 (1.21–5.79)	2.15 (0.94–4.89)	1.80 (0.75–4.33)	1.68 (0.69–4.08)
First born		5.01 (3.18–7.89)	4.62 (2.86–7.47)	4.29 (2.67–6.88)	*4.16 (2.52–6.86)*
Previous history of stillbirth		0.91 (0.37–2.26)^‡^			
Previous history of miscarriage		1.12 (0.67–1.86)^‡^			
Maternal history of diabetes mellitus irrespective of pregnancy		1.50 (0.18–12.79)^‡^			
Maternal history of hypertension irrespective of pregnancy		1.33 (0.38–4.60)^‡^			
No maternal antenatal care visit during pregnancy			1.87 (1.19–2.91)	2.34 (1.51–3.63)	*1.99 (1.25–3.16)*
Mother did not receive 2 tetanus toxoid injections during pregnancy			1.93 (1.25–2.99)	1.74 (1.12–2.70)	1.47 (0.92–2.35)
Mother did not consume iron folic acid tablets during pregnancy			1.16 (0.77–1.76)^‡^		
Pregnancy with multiple foetuses			1.36 (0.51–3.67)^‡^		
Maternal high blood pressure in the last trimester of pregnancy			1.09 (0.38–3.17)^‡^		
Mother had malaria in the last trimester of pregnancy			1.16 (0.34–3.94)^‡^		
Mother diagnosed with syphilis during this pregnancy					
No			1.00		
Yes			3.58 (0.47–27.16)^‡^		
Do not know			1.57 (0.81–3.04)^‡^		
Mother had fever in the last 3 months of pregnancy			0.92 (0.54–1.56)^‡^		
Mother had convulsions in the last 3 months of pregnancy			1.33 (0.77–2.29)^‡^		
Mother was informed that the baby was not growing adequately inside the womb			1.70 (0.83–3.49)	1.38 (0.69–2.77)^‡^	
Gestation period					
7 months			18.58 (10.30–33.52)	20.74 (11.42–37.67)	*18.27 (9.61–34.74)*
> 7–8 months			17.00 (10.42–27.71)	17.25 (10.61–28.04)	*15.56 (9.27–26.12)*
> 8 months			1.00	1.00	1.00
Deferred delivery				4.72 (1.88–11.82)	*5.61 (2.23–14.13)*
Spontaneous labour				2.17 (1.47–3.20)	*2.14 (1.40–3.28)*
Foul smelling discharge				2.45 (1.40–4.26)	*2.04 (1.10–3.78)*
Place of delivery					
Public facility					1.00
Private facility					*2.00 (1.15–3.46)*
Home					*2.44 (1.51–3.95)*
Vaginal delivery					*2.78 (1.20–6.45)*
Push/forceful pull done during delivery by the health provider					*4.06 (2.39–6.89)*
Entangled cord around the baby’s neck					
No					1.00
Yes					1.35 (0.62–2.94)
Do not know					0.37 (0.13–1.07)
Breech presentation of the baby					1.82 (0.94–3.52)
Intrapartum stillbirth					
Rural place of residence	1.33 (0.57–3.12)	1.25 (0.54–2.92)	1.13 (0.48–2.65)	1.15 (0.49–2.69)	0.97 (0.41–2.31)
Boy baby	1.16 (0.78–1.74)	1.20 (0.79–1.81)	1.39 (0.90–2.15)	1.33 (0.86–2.05)	1.20 (0.77–1.85)
Wealth index quartile					
I	1.20 (0.66–2.15)^‡^				
II	1.38 (0.78–2.45)^‡^				
III	0.87 (0.47–1.64)^‡^				
IV	1.00				
Maternal age					
15–19 years		1.00	1.00	1.00	1.00
20–24 years		1.13 (0.47–2.69)	1.26 (0.49–3.24)	1.29 (0.50–3.32)	1.27 (0.49–3.29)
25–29 years		2.35 (0.92–5.99)	2.83 (1.03–7.76)	3.16 (1.15–8.67)	*3.33 (1.20–9.25)*
≥ 30 years		2.47 (0.84–7.23)	3.18 (1.02–9.93)	3.50 (1.12–10.96)	2.73 (0.83–8.93)
Solid cooking fuel use		1.59 (0.99–2.55)	1.73 (1.05–2.86)	1.70 (1.03–2.79)	*1.70 (1.01–2.88)*
Any tobacco use ever		0.40 (0.06–2.92)^‡^			
First born		5.85 (3.50–9.76)	6.30 (3.62–10.94)	6.87 (3.98–11.85)	*7.22 (4.09–12.76)*
Previous history of stillbirth		2.33 (1.15–4.72)	2.41 (1.18–4.91)	2.27 (1.11–4.65)	1.92 (0.90–4.07)
Previous history of miscarriage		1.37 (0.79–2.36)^‡^			
Maternal history of diabetes mellitus irrespective of pregnancy		4.16 (0.78–22.09)	5.73 (1.22–27.01)	4.50 (0.99–20.36)	3.03 (0.63–14.55)
Maternal history of high blood pressure irrespective of pregnancy		1.32 (0.35–5.05)^‡^			
No maternal antenatal care visit during pregnancy			0.91 (0.49–1.68)^‡^		
Mother did not receive 2 tetanus toxoid injections during pregnancy			0.92 (0.49–1.70)^‡^		
Mother did not consume iron folic acid tablets during pregnancy			1.14 (0.73–1.79)^‡^		
Pregnancy with multiple foetuses			3.36 (1.16–9.72)	3.36 (1.17–9.70)	2.73 (0.91–8.21)
Maternal hypertension in the last trimester of pregnancy			0.74 (0.17–3.32)^‡^		
Mother had malaria in the last trimester of pregnancy			0.65 (0.14–2.95)^‡^		
Mother diagnosed with syphilis during this pregnancy					
No			1.00		
Yes			Empty		
Do not know			0.87 (0.34–2.21)^‡^		
Mother had fever in the last 3 months of pregnancy			1.62 (0.99–2.66)	1.43 (0.86–2.37)	1.11 (0.66–1.89)
Mother had convulsions in the last 3 months of pregnancy			0.37 (0.13–1.05)	0.48 (0.19–1.20)	*0.38 (0.15–0.97)*
Mother was informed that the baby was not growing adequately inside the womb			1.29 (0.49–3.39)^‡^		
Gestation period					
7 months			6.92 (2.84–16.88)	7.55 (3.15–18.10)	*5.31 (2.02–13.91)*
> 7–8 months			4.16 (1.86–9.27)	4.11 (1.85–9.15)	*4.14 (1.90–9.04)*
> 8 months			1.00	1.00	1.00
Deferred delivery				4.04 (1.40–11.67)	3.03 (0.97–9.46)
Spontaneous labour				1.04 (0.65–1.67)^‡^	
Foul smelling discharge				0.99 (0.41–2.41)^‡^	
Place of delivery					
Public facility					1.00
Private facility					1.28 (0.68–2.43)
Home					*2.05 (1.23–3.40)*
Vaginal delivery					1.08 (0.52–2.23)
Push/forceful pull done during delivery by the health provider					*4.36 (2.49–7.63)*
Entangled cord around baby’s neck					
No					1.00
Yes					1.00 (0.41–2.47)
Do not know					0.24 (0.06–1.02)
Breech presentation of the baby					*4.33 (2.38–7.89)*

*Model adjusted for sex of the baby and place of residence

^‡^*p* ≥ 0.2, hence excluded from the sequential model

## Discussion

To our knowledge, this study is the first large-scale state-wide population-based assessment of all births to facilitate understanding of epidemiology of stillbirths by type in India. The stillbirth rate was 15.4 per 1000 births for Bihar state with no statistically significant difference between boys and girls. In addition to demographic and clinical risk factors for antepartum and intrapartum stillbirths, this study has documented the increased risk of stillbirths in deferred and referred deliveries highlighting aspects of health care around labour and delivery that need attention in addition to improved skills of health providers to reduce stillbirths.

The smallest babies, in terms of gestational age and birthweight, are known to be at the highest risk of stillbirth [18, 19]. In this study, gestation period of ≤ 8 months was the strongest risk factor for both antepartum and intrapartum stillbirths, and particularly high for antepartum stillbirths. A total of 2.9% of all babies and 29.1% of stillborn babies had gestation period of ≤ 8 months in our study. As the pregnancy length was captured in months in our study, it is not possible for us to comment on whether the babies with gestation period of ≤ 8 months were very or moderately pre-term [9]. Pre-term labour is considered to be a syndrome initiated by multiple mechanisms, and more understanding in classification of pre-term and of its risk factors is needed [20–22]. Our data do not allow us to distinguish between pre-term labour as an increased risk for stillbirth or if a condition that caused stillbirth precipitated the early delivery, but these highlight that in addition to improving data on gestational age, differentiating spontaneous and medically induced pre-term births is of relevance to INAP to reduce adverse birth outcomes with rising caesarean section rate in India [23].

Antepartum deaths accounted for half of the stillbirths in this population, as reported previously and elsewhere [3, 18]. The women with deferred births were fivefold more likely to have an antepartum stillbirth. The major reason for them to be sent back from the health facility was that the health provider thought there was still time for delivery. Also, women who reported spontaneous labour and foul smelling discharge were significantly more likely to have antepartum stillbirth. We did not document why these women thought they were ready to deliver when they first visited the health facility for delivery. We did not document the frequency of foetal movements for all births or whether these were checked by the health provider before deferring the delivery; only the absence of foetal movements in stillborn was documented. It is well established that reduced foetal movement is associated with increased risk of poor pregnancy outcomes such as foetal growth restriction and stillbirth [24–26] and that maternal perception of decreased foetal movements is associated with adverse pregnancy outcomes, including stillbirth [26, 27]. Also, a sudden increase in vigorous foetal movements indicating foetal compromise possibly due to an hypoxic-ischaemic insult is also reported [26]. Monitoring of foetal movements at every antenatal care visit and teaching the pregnant women how to monitor foetal movements are recommended in the Indian health worker guidelines for delivery care [28]; however, not much is known about implementation of these in practice. To strengthen surveillance mechanism to prevent stillbirths in INAP [1], further research is needed to understand what methods to monitor are appropriate, what the women are informed about the foetal movements by health providers [29–32], the process of foetal heart rate monitoring in medical records at health facilities, and on what basis the health providers defer a delivery.

Foetal growth restriction is known to be associated with antepartum stillbirth [33–35]. Poor foetal growth and baby small-for-gestational age at birth were associated with antepartum stillbirth in the INTERGROWTH-21 Project [16]. We did not find a significant association of stillbirth with women being informed that the baby was not growing adequately inside the womb. We are unable to comment on birthweight as most of the stillborn babies were not weighed at birth as also reported by us previously [3]. However, of importance to note in this study is that only 23.7% of the livebirths were not weighed as compared with 84.6% of the stillbirths. Furthermore, the size of the baby in this study did not necessarily corroborate with birthweight as nearly 70% of the babies who were reported to be not growing adequately inside the womb weighed ≥ 2 kg at birth. Although the Indian government guidelines on the newborn care facilities include weighing of the newborn at birth [36], it is clear that those stillborn are not weighed. Also, weighing newborn is currently not included in the health worker guidelines for delivery care [28]. As the INAP envisages strengthening surveillance mechanism for stillbirths [1], it is imperative for these guidelines explicitly state that every newborn should be weighed irrespective of the baby’s live status at birth.

Pregnancies with no ANC were associated with a higher risk of antepartum stillbirths and with borderline significance for overall stillbirths. ANC is meant to deliver maternal interventions to reduce adverse outcomes by preventing, identifying, or treating infections, pregnancy-induced conditions, and undernutrition [33, 37, 38]. Syphilis and malaria are among the largest contributors to stillbirths of infectious causes in the low-income countries [39–41], whereas placental conditions, pregnancy-induced hypertension, and congenital anomalies contribute to the antepartum deaths globally [40, 42]. Considering all births in our population, syphilis, malaria, and pregnancy-induced hypertension were reported only by 0.4%, 2.3%, and 2.6% women. However, based on our experience, it is important to note here that negative reporting does not necessarily mean that these conditions did not exist. It includes a variety—women who were checked and informed that they did not have a given condition, those who were checked but not informed whether they had or did not have the condition, women who were not checked for these conditions, and women who did not know if they were checked for these conditions. Among the 81.2% women who reported at least one ANC visit in this study, 48%, 46.9%, and 31.1% were not asked to give blood sample, give urine sample, or have their blood pressure checked even once, respectively. All these point to inadequacy of antenatal care, and significant reduction in antepartum stillbirths is unlikely in India unless INAP can ensure improved quality of ANC services to deliver maternal interventions in addition to improved coverage.

We found evidence of sub-optimal quality of care around labour and delivery as babies presenting in breech position, pregnancies with multiple foetuses, and deliveries with “push and forceful pull” performed during the delivery by the health provider had a significantly higher odds of stillbirth, both for antepartum and intrapartum stillbirths. Furthermore, antepartum stillbirth was more likely to be delivered vaginally. Emergency obstetric care including caesarean section is highly effective in reducing stillbirths [33, 43, 44]; however, as we have previously highlighted, some health providers in obstructed deliveries and/or prolonged labour continue to wait for a vaginal delivery instead of opting for a timely caesarean section that could possibly reduce the chance of stillbirth in some cases [3]. Better quality of care during labour and childbirth in deliveries with complications is likely to result in the highest number of stillbirths averted [37]. With intrapartum stillbirths accounting for 40% of all stillbirths in this population, improving quality of care during labour and childbirth as proposed in the INAP should facilitate notable reduction in intrapartum deaths [1].

Place of delivery was an important risk factor in determining stillbirth as an outcome. Though the proportion of home deliveries in this population has declined significantly by 20.5% over the last 5 years [7], 30% of the women still delivered at home with these deliveries having significantly higher odds of stillbirths. As the home deliveries conducted by untrained birth attendants (such as untrained Dai, family member, and friends) had a higher risk of stillbirth as compared with a skilled birth attendant, it is important to understand birth preparedness in addition to encouraging safe facility deliveries [45–47]. The private sector facilities also had a higher odds of stillbirth than public sector facilities, antepartum in particular. As highlighted by this study, this finding needs to be interpreted within the context of referral of deliveries from one facility to another, both within and between public and private facilities [48]. In general irrespective of the place of delivery, the high odds of stillbirth for deliveries with breech position of the baby and those that included push/forceful pull of the baby by the health provider highlight the need for skill-building of health providers to not only identify and manage complications, but also to do timely referral [1, 28, 49]. Majority of the deliveries with breech position of the baby (75.4%) were referred by one health provider to another in this study. Also, pregnancies with multiple foetuses had significantly higher odds of stillbirth than singleton pregnancies [16]. We did not document the time of death of the babies, but we have previously highlighted in this population the reluctance of health providers to deliver a dead baby which also results in referral for delivering a dead baby [3]. Poor quality obstetric referral services, higher odds of adverse birth outcomes in referral deliveries, and low competence of health staff providing emergency obstetric care in India have been documented previously [48–52]. INAP recognises the shortage of appropriately trained human resources as a major bottleneck in improving quality of care in addition to poor quality of care, and recommends effective referral mechanisms for complicated deliveries [1]. As indicated by these findings, improved monitoring and evaluation practices are necessary along with better understanding of how referrals work to inform implementation of effective referral mechanisms [48, 53, 54]. With private sector dominating provision of emergency obstetric care in India [55, 56], INAP needs to encourage partnership with private sector for improved quality of care in addition to enforcing regulations on private sector [1, 57].

The finding of greater risk of stillbirths with higher maternal age and in first born has been reported from many developing countries [18, 58, 59]. Boy babies are reported to at a 10% higher risk of stillbirths than girl babies [60], and 62% of stillborn were boys in our previous assessment which included only stillbirths [3]. Though boys accounted for 56.4% of all stillbirths in this study, we did not find the SBR to be different for boys and girls when considering all births. It is important to note that the odds of stillbirth were significantly higher for boys in logistic regression until model 4 and then lost statistical significance in the last model on including delivery-related risk factors. This finding needs to be explored further. Solid cooking fuel use had a slight but significant association with intrapartum stillbirth, and such an association with stillbirth has been reported recently from rural communities including India [61].

There are some limitations to the study findings. As is the case with surveys, the findings should be interpreted within the context of recall bias of the respondent. The gestational age was captured in months instead of weeks as the pregnancy length in India is reported in months. The last menstrual period forms the basis for most and is considered a reliable estimate for measuring gestational age in both developing and developed country settings [62, 63]. To classify stillbirths as antepartum or intrapartum, a previously used cut-off of within 8 h since the last felt baby’s movement was considered [3, 10–12], and we preferred the baby’s movement over the description of stillborn baby because appearance is reported to be a less accurate proxy for death-to-delivery interval [11, 13, 14]. If we had given preference to stillborn baby’s description over movement, 51 (18.5%) cases would then be classified as antepartum and 96 (34.9%) cases as intrapartum deaths. INAP should encourage documentation of accurate time of delivery and that of the baby’s demise where possible in medical records at health facilities to differentiate antepartum and intrapartum stillbirths, and should establish mortality audit to address breakdowns in clinical care or processes to strengthen stillbirth surveillance [64].

There are several strengths of this study. Some previously available population level reports on stillbirths have been either been on stillbirths only, case-control in nature, small sample size, and/or from a select city/area [40, 65, 66]. This study’s strength is the large-scale state-wide representative data on all births that allows for detailed understanding of epidemiology of stillbirths by type. We aimed to generate robust estimates for all births and stillbirths by documenting all in/out migration among the reproductive age women who had a pregnancy outcome in the period of interest that provides an appropriate denominator for the stillbirth estimation. We strengthened the numerator for these estimates by confirming stillbirth at three points in time from enumeration through the analysis by confirming signs of life, which is different from the DHS surveys which do not confirm signs of life from the respondent when documenting a stillbirth [1, 3]. We used a hierarchical approach to the risk factor analysis that gave importance to distal determinants of stillbirths, and have presented results in a manner that allows for better understanding of association of various risk factors of interest [16].

On comparing the SBR in this study with that in 2012 [3], a reduction of 27.4% (95% CI 12.9–41.8) is seen in Bihar over 4 years. As we do not have comparative data on the coverage of services for these years on all births in 2012 survey, it is difficult for us to comment on the possible reasons for this reduction. However, comparing data on livebirths in the same population from this study and a previous survey in 2011 [7], there has been a significant increase in coverage of antenatal care and public sector delivery (10.2% and 18.8%, respectively; *z* test *p* < 0.001) and decrease in home deliveries (20.5%; *z* test *p* < 0.001) in this population. Improvements in intrapartum practices through nurse mentoring are also documented in the Bihar Technical Support Programme [67]. These could have possibly played a role in stillbirth reduction in this population.

## Conclusions

**Fig. 3:**
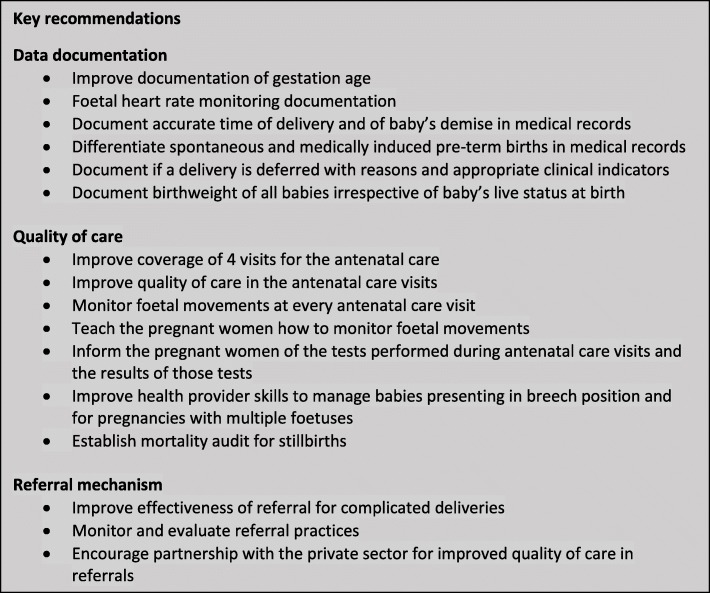
Key recommendations for the India National Action Plan (INAP) to strengthen surveillance mechanism for stillbirths in addition to those proposed in the INAP

This study provides a population-based understanding of stillbirths from a large number of births from the state of Bihar highlighting the risk factors that need to be addressed to reduce the burden of stillbirths. The INAP is well placed to utilise the findings of this study to further strengthen its approach to meet the stillbirth reduction target by 2030 (Fig. 3). It may be prudent for INAP to explicitly state and promote specific strategies to reduce antepartum stillbirths in addition to intrapartum deaths.

## Additional files

Additional file 1: **Table S1.** Basic descriptive for risk factors during labour and delivery for all births including stillbirths by the place of delivery between January and December 2016 in the Indian state of Bihar. (DOCX 16 kb)

Additional file 2: **Table S2.** Results of multiple logistic regression for association of stillbirth for sub-group analysis for deferred deliveries with select risk factors in the Indian state of Bihar. (DOCX 12 kb)

Additional file 3: **Table S3.** Distribution of the health provider who delivered the baby for all births, and for facility births based on referral between January and December 2016 in the Indian state of Bihar. (DOCX 13 kb)

Additional file 4: **Table S4.** Results of sequential multiple logistic regression models for association of stillbirths in facility deliveries with inclusion of referred delivery as a risk factor along with other risk factors in the Indian state of Bihar. Statistically significant odds ratios are shown in bold in the final model 5. (DOCX 16 kb)

Additional file 5: **Table S5.** Results of multiple logistic regression for association of stillbirth for sub-group analysis for referred deliveries with select risk factors in the Indian state of Bihar. (DOCX 12 kb)

## Abbreviations

INAP: Indian Newborn Action Plan
OR: Odds ratio
SBR: Stillbirth rate
