# Supplementary material for: Deferred and referred deliveries contribute to stillbirths in the Indian state of Bihar: results from a population-based survey of all births
Source: BMC Med. 2019 Feb 7;17:28. doi: 10.1186/s12916-019-1265-1 (PMC6366028; doi:10.1186/s12916-019-1265-1)
Supplement: Supplementary file 1 — Table S1. Basic descriptive for risk factors during labour and delivery for all births including stillbirths by the place of delivery between January and December 2016 in the Indian state of Bihar. (DOCX 16 kb) [file 12916_2019_1265_MOESM1_ESM.docx]

**Additional Table 1. Basic descriptive for risk factors during labour and delivery for all births including stillbirths by the place of delivery between January and December 2016 in the Indian state of Bihar.**

| **Risk factor** |  | **Public facility** | | **Private facility** | | **Home** | |
| --- | --- | --- | --- | --- | --- | --- | --- |
|  |  | **All births**  **N=10,712 (%)** | **Stillbirths**  **N=89 (%)** | **All births**  **N=3,394 (%)** | **Stillbirths**  **N=80 (%)** | **All births**  **N=6,023 (%)** | **Stillbirths**  **N=101 (%)** |
| Mother had come for delivery earlier but was asked to come later for delivery (deferred delivery)^*^ | Yes | 74 (0.7%) | 6 (6.7%) | 71 (2.1%) | 3 (3.8%) | 29 (0.5%) | 6 (6.0%) |
|  | No | 10,537 (99.3%) | 83 (93.3%) | 3,282 (97.9%) | 77 (96.3%) | 5,933 (99.5%) | 94 (94.0%) |
| Ultrasound done during labour^†^ | Yes | 382 (3.6%) | 9 (10.1%) | 550 (16.5%) | 29 (36.3%) | 73 (1.2%) | 5 (5.0%) |
|  | No | 10,177 (96.4%) | 80 (89.9%) | 2,794 (83.6%) | 51 (63.8%) | 5,862 (98.8%) | 96 (95.1%) |
| Spontaneous labour^‡^ | Yes | 7,647 (71.6%) | 51 (59.3%) | 1,999 (60.2%) | 28 (38.9%) | 5,143 (85.8%) | 85 (85.9%) |
|  | No | 3,033 (28.4%) | 35 (40.7%) | 1,321 (39.8%) | 44 (61.1%) | 853 (14.2%) | 14 (14.1%) |
| Foul smelling discharge^§^ | Yes | 537 (5.1%) | 11 (12.4%) | 187 (5.6%) | 9 (11.3%) | 280 (4.7%) | 8 (7.9%) |
|  | No | 10,106 (95.0%) | 78 (87.6%) | 3,179 (94.4%) | 71 (88.8%) | 5,707 (95.3%) | 93 (92.1%) |
| Who delivered the baby^#^ | Doctor | 1,237 (11.6%) | 16 (18.2%) | 1,867 (55.2%) | 50 (63.3%) | 94 (1.6%) | 1 (1.0%) |
|  | Nurse | 7,565 (70.8%) | 53 (60.2%) | 1,326 (39.2%) | 25 (31.7%) | 178 (3.0%) | 0 (0.0%) |
|  | ANM/Skilled birth attendant | 1,671 (15.7%) | 12 (13.6%) | 148 (4.4%) | 1 (1.3%) | 122 (2.0%) | 0 (0.0%) |
|  | Untrained Dai | 41 (0.4%) | 1 (1.1%) | 8 (0.2%) | 0 (0.0%) | 3,989 (66.3%) | 52 (52.5%) |
|  | Others | 166 (1.6%) | 6 (6.8%) | 36 (1.1%) | 3 (3.8%) | 1,630 (27.1%) | 46 (46.5%) |
| Vaginal delivery^**^ | Yes | 10,118 (94.5%) | 78 (89.7%) | 1,947 (57.5%) | 50 (64.1%) | 6,013 (100%) | 100 (100.0%) |
|  | No | 586 (5.5%) | 9 (10.3%) | 1,441 (42.5%) | 28 (35.9%) | 0 |  |
| Push/ forceful pull done during delivery by the health provider^††^ | Yes | 569 (5.4%) | 25 (29.4%) | 312 (9.7%) | 21 (27.6%) | 143 (2.4%) | 14 (13.9%) |
|  | No | 9,977 (94.6%) | 60 (70.6%) | 2,898 (90.3%) | 55 (72.4%) | 5,844 (97.6%) | 87 (86.1%) |
| Entangled cord around baby’s neck^‡‡^ | Yes | 377 (3.5%) | 10 (11.2%) | 139 (4.1%) | 5 (6.3%) | 266 (4.4%) | 6 (5.9%) |
|  | Don’t know | 978 (9.1%) | 5 (5.6%) | 398 (11.7%) | 8 (10.0%) | 387 (6.4%) | 2 (2.0%) |
|  | No | 9,347 (87.3%) | 74 (83.2%) | 2,853 (84.2%) | 67 (83.8%) | 5,367 (89.2%) | 93 (92.1%) |
| Breech position of the baby^§§^ | Yes | 342 (3.2%) | 18 (20.9%) | 155 (4.8%) | 7 (9.3%) | 174 (2.9%) | 14 (13.9%) |
|  | No | 10,236 (96.8%) | 68 (79.1%) | 3,054 (95.2%) | 68 (90.7%) | 5,804 (97.1%) | 87 (86.1%) |
| Birthweight of the baby (kilograms)^##^ | ≥2.0 | 9,074 (85.4%) | 19 (22.4%) | 2,615 (78.3%) | 15 (20.3%) | 1,197 (20.1%) | 2 (2.1%) |
|  | <2.0 | 365 (3.4%) | 2 (2.4%) | 159 (4.8%) | 1 (1.4%) | 57 (1.0%) | 0 (0.0%) |
|  | Not weighed | 322 (3.0%) | 0 (0.0%) | 279 (8.4%) | 0 (0.0%) | 4,100 (68.8%) | 0 (0.0%) |
|  | Don’t know if weighed | 861 (8.1%) | 64 (75.3%) | 289 (8.7%) | 58 (78.4%) | 608 (10.2%) | 93 (97.9%) |

^*^Data not available for 101, 41 and 61 births in public facility, private facility and home delivery, respectively; Chi-square test for significance: p<0.001 for public facility and home delivery, and 0.305 for private facility

^†^Data not available for 153, 50 and 88 births in public facility, private facility and home delivery, respectively; Chi-square test for significance: p=0.001 for public facility and home delivery, and <0.001 for private facility

^‡^Data not available for 32, 74 and 27 in births in public facility, private facility and home delivery, respectively; Chi-square test for significance: p=0.011 for public, <0.001 for private, and 0.981 for home delivery

^§^Data not available for 69, 28 and 36 births in public facility, private facility and home delivery, respectively; Chi-square test for significance: p=0.002 for public, 0.024 for private and 0.119 for home delivery

^#^Data not available for 32, 9 and 10 births in public facility, private facility and home delivery, respectively; others in who delivered the baby include unskilled birth attendants, unqualified doctor, family member, neighbour, friend, none; Chi-square test for significance: p<0.001 for public and home delivery and 0.044 for private facility

^**^Data not available for 8, 6 and 10 births in public facility, private facility and home delivery, respectively; Chi-square test for significance: p=0.045 for public, 0.23 for private facility

^††^Data not available for166, 184 and 36 births in public facility, private facility and home delivery, respectively; Chi-square test for significance: p<0.001 for all

^‡‡^Data not available for 10, 4 and 3 births in public facility, private facility and home delivery, respectively; Chi-square test for significance: p<0.001 for public, 0.567 for private and 0.150 for home delivery

^§§^Data not available for 134, 185 and 45 births in public facility, private facility and home delivery, respectively; Chi-square test for significance: p<0.001 for public facility and home delivery and 0.066 for private facility

^##^ Data not available for 90, 52 and 61 births in public facility, private facility and home delivery, respectively; Chi-square test for significance: p<0.001 for all
