# Supplementary material for: Deferred and referred deliveries contribute to stillbirths in the Indian state of Bihar: results from a population-based survey of all births
Source: BMC Med. 2019 Feb 7;17:28. doi: 10.1186/s12916-019-1265-1 (PMC6366028; doi:10.1186/s12916-019-1265-1)
Supplement: Supplementary file 2 — Table S2. Results of multiple logistic regression for association of stillbirth for sub-group analysis for deferred deliveries with select risk factors in the Indian state of Bihar. (DOCX 12 kb) [file 12916_2019_1265_MOESM2_ESM.docx]

**Additional Table 2. Results of multiple logistic regression for association of stillbirth for sub-group analysis for deferred deliveries with select risk factors in the Indian state of Bihar.**

|  |  | **Adjusted odds ratio for stillbirth**  **(95% confidence interval)*** |
| --- | --- | --- |
| **By place of delivery** | | |
| Deferred delivery | Home delivery |  |
| No | No | 1.00 |
| Yes | No | 5.68 (2.84-11.37) |
| No | Yes | 1.35 (1.05-1.75) |
| Yes | Yes | 23.25 (9.31-58.06) |
| **By breech presentation of the baby** | | |
| Deferred delivery | Breech presentation of the baby |  |
| No | No | 1.00 |
| Yes | No | 7.27 (3.87-13.64) |
| No | Yes | 5.25 (3.66-7.51) |
| Yes | Yes | 23.09 (7.58-70.31) |
| **By push and forceful pull done by health provider during delivery** | | |
| Deferred delivery | Push and forceful pull done by health provider during delivery |  |
| No | No | 1.00 |
| Yes | No | 5.65 (2.73-11.70) |
| No | Yes | 5.28 (3.87-7.20) |
| Yes | Yes | 43.80 (17.46-109.89) |

*Adjusted for place of residence and sex of the baby
