# Supplementary material for: Deferred and referred deliveries contribute to stillbirths in the Indian state of Bihar: results from a population-based survey of all births
Source: BMC Med. 2019 Feb 7;17:28. doi: 10.1186/s12916-019-1265-1 (PMC6366028; doi:10.1186/s12916-019-1265-1)
Supplement: Supplementary file 3 — Table S3. Distribution of the health provider who delivered the baby for all births, and for facility births based on referral between January and December 2016 in the Indian state of Bihar. (DOCX 13 kb) [file 12916_2019_1265_MOESM3_ESM.docx]

**Additional Table 3. Distribution of the health provider who delivered the baby for all births, and for facility births based on referral between January and December 2016 in the Indian state of Bihar.**

| **Health provider who delivered the baby** | **All births**^†^  **N=20,152** | | **Referred births**^‡^  **N=827** | | **Non-referred births**^§^  **N=13,275** | |
| --- | --- | --- | --- | --- | --- | --- |
|  | **Total**  **(N, %)** | **Stillbirths**  **(% of N)** | **Total**  **(N, %)** | **Stillbirths**  **(% of N)** | **Total**  **(N, %)** | **Stillbirths**  **(% of N)** |
| Doctor | 3,202 (15.9%) | 67  (2.09%) | 459 (55.5%) | 30  (6.54%) | 2,645 (19.9%) | 36  (1.36%) |
| Nurse | 9,080 (45.1%) | 78  (0.86%) | 315 (38.1%) | 15  (4.76%) | 8,576 (64.6%) | 63  (0.73%) |
| ANM/skilled birth attendant | 1,943 (9.7%) | 14  (0.72%) | 42  (5.1%) | 1  (2.38%) | 1,777 (13.4%) | 12  (0.68%) |
| Untrained Dai | 4,039 (20.1%) | 53  (1.31%) | 2  (0.2%) | 0 | 47  (0.4%) | 1  (2.13%) |
| Others* | 1,833 (9.1%) | 55  (3.00%) | 7  (0.9%) | 0 | 195  (1.5%) | 9  (4.62%) |
| Don’t know | 46  (0.2%) | 1  (2.17%) | 2  (0.2%) | 0 | 35  (0.3%) | 0 |

*Unskilled birth attendants, unqualified doctor, family member, neighbour, friend, none

^†^Data not available for 9 births on type of health provider, and 4 women on whether they were referred or not; Chi-square test for significance: p<0.001

^‡^Births at home are excluded; Chi-square test for significance: p=0.751

^§^Births at home are excluded; Chi-square test for significance: p<0.000
