# Supplementary material for: Deferred and referred deliveries contribute to stillbirths in the Indian state of Bihar: results from a population-based survey of all births
Source: BMC Med. 2019 Feb 7;17:28. doi: 10.1186/s12916-019-1265-1 (PMC6366028; doi:10.1186/s12916-019-1265-1)
Supplement: Supplementary file 4 — Table S4. Results of sequential multiple logistic regression models for association of stillbirths in facility deliveries with inclusion of referred delivery as a risk factor along with other risk factors in the Indian state of Bihar. Statistically significant odds ratios are shown in bold in the final model 5. (DOCX 16 kb) [file 12916_2019_1265_MOESM4_ESM.docx]

**Additional Table 4. Results of sequential multiple logistic regression models for association of stillbirths in facility deliveries with inclusion of referred delivery as a risk factor along with other risk factors in the Indian state of Bihar. Statistically significant odds ratios are shown in bold in the final model 5.**

| **Risk factor** | **Adjusted odds ratio for stillbirth (95% confidence interval)** | | | | |
| --- | --- | --- | --- | --- | --- |
|  | **Model 1*** | **Model 2*** | **Model 3*** | **Model 4*** | **Model 5*** |
| **Rural place of residence** | 1.52 (0.81-2.85) | 1.64 (0.88-3.07) | 1.61 (0.85-3.05) | 1.65 (0.87-3.12) | 1.41 (0.71-2.80) |
| **Boy baby** | 1.31 (0.96-1.79) | 1.37 (1.00-1.88) | 1.55 (1.11-2.17) | 1.55 (1.10-2.18) | **1.43 (1.00-2.05)** |
| **Wealth index quartile** |  |  |  |  |  |
| *I* | 1.02 (0.64-1.61) ^‡^ |  |  |  |  |
| *II* | 1.10 (0.71-1.71) ^‡^ |  |  |  |  |
| *III* | 1.20 (0.79-1.81) ^‡^ |  |  |  |  |
| *IV* | 1.00 |  |  |  |  |
| **Maternal age** |  |  |  |  |  |
| *15-19 years* |  | 1.00 | 1.00 | 1.00 | 1.00 |
| *20-24 years* |  | 1.29 (0.68-2.44) | 1.18 (0.61-2.26) | 1.25 (0.63-2.47) | 1.29 (0.64-2.60) |
| *25-29 years* |  | 1.74 (0.85-3.55) | 1.60 (0.77-3.31) | 1.68 (0.79-3.57) | 1.80 (0.82-3.96) |
| *>=30 years* |  | 2.19 (0.96-4.98) | 1.98 (0.86-4.58) | 2.02 (0.85-4.82) | 1.63 (0.64-4.18) |
| **Solid cooking fuel use** |  | 0.90 (0.65-1.24) ^‡^ |  |  |  |
| **Any tobacco use ever** |  | 1.62 (0.65-4.05) ^‡^ |  |  |  |
| **First born** |  | 4.62 (3.14-6.79) | 4.93 (3.25-7.48) | 4.61 (3.02-7.03) | **4.44 (2.82-7.01)** |
| **Previous history of stillbirth** |  | 1.17 (0.57-2.44) ^‡^ |  |  |  |
| **Previous history of miscarriage** |  | 1.19 (0.77-1.82) ^‡^ |  |  |  |
| **Maternal history of diabetes mellitus irrespective of pregnancy** |  | 2.86 (0.75-10.88) | 3.23 (0.87-12.03) | 3.20 (0.90-11.34) | 2.39 (0.61-9.43) |
| **Maternal history of high blood pressure irrespective of pregnancy** |  | 1.64 (0.65-4.15) ^‡^ |  |  |  |
| **No maternal antenatal care visit during pregnancy** |  |  | 1.26 (0.77-2.04) ^‡^ |  |  |
| **Mother did not receive 2 tetanus toxoid injections during pregnancy** |  |  | 2.03 (1.36-3.04) | 1.81 (1.19-2.75) | **1.82 (1.16-2.86)** |
| **Mother did not consume Iron Folic Acid tablets during pregnancy** |  |  | 1.08 (0.77-1.52) ^‡^ |  |  |
| **Pregnancy with multiple foetuses** |  |  | 1.92 (0.83-4.45) | 2.45 (1.06-5.65) | 1.51 (0.60-3.81) |
| **Maternal hypertension in the last trimester of pregnancy** |  |  | 1.83 (0.86-3.88) | 1.90 (0.94-3.82) | 1.63 (0.78-3.37) |
| **Mother had malaria in the last trimester of pregnancy** |  |  | 1.09 (0.40-3.00) ^‡^ |  |  |
| **Mother were diagnosed with syphilis during this pregnancy** |  |  |  |  |  |
| **Yes** |  |  | Empty |  |  |
| **Don’t know** |  |  | 1.09 (0.59-2.02) ^‡^ |  |  |
| **Mother had fever in the last 3 months of pregnancy** |  |  | 1.01 (0.66-1.56) ^‡^ |  |  |
| **Mother had convulsions in the last 3 months of pregnancy** |  |  | 0.83 (0.48-1.43) ^‡^ |  |  |
| **Mother informed baby was not growing adequately inside the womb** |  |  | 1.80 (0.99-3.26) | 1.50 (0.84-2.68) | 1.50 (0.82-2.73) |
| **Gestation period** |  |  |  |  |  |
| *7 months* |  |  | 21.02 (12.35-35.80) | 20.62 (11.89-35.75) | **16.77 (8.91-31.59)** |
| *>7-8 months* |  |  | 7.81 (4.68-13.03) | 5.96 (3.41-10.44) | **5.70 (3.12-10.43)** |
| *>8 months* |  |  | 1.00 | 1.00 | 1.00 |
| **Deferred delivery** |  |  |  | 4.21 (1.91-9.27) | **2.74 (1.17-6.41)** |
| **Spontaneous labour** |  |  |  | 1.86 (1.33-2.61) | **1.66 (1.15-2.37)** |
| **Foul smelling discharge** |  |  |  | 1.49 (0.85-2.62) | 1.21 (0.65-2.27) |
| **Referred delivery** |  |  |  |  | **3.32 (2.03-5.43)** |
| **Place of delivery** |  |  |  |  |  |
| *Public facility* |  |  |  |  | 1.00 |
| *Private facility* |  |  |  |  | 1.40 (0.93-2.11) |
| **Vaginal delivery** |  |  |  |  | **1.91 (1.16-3.16)** |
| **Push/ forceful pull done during delivery by the health provider** |  |  |  |  | **4.13 (2.72-6.28)** |
| **Entangled cord** |  |  |  |  |  |
| *No* |  |  |  |  | 1.00 |
| *Yes* |  |  |  |  | 1.66 (0.87-3.14) |
| *Don’t know* |  |  |  |  | **0.41 (0.17-0.97)** |
| **Breech presentation of the baby** |  |  |  |  | **2.31 (1.32-4.04)** |

*Model adjusted for sex of the baby and place of residence

^‡^P>0.2, and hence excluded from the sequential model
