# Supplementary material for: Deferred and referred deliveries contribute to stillbirths in the Indian state of Bihar: results from a population-based survey of all births
Source: BMC Med. 2019 Feb 7;17:28. doi: 10.1186/s12916-019-1265-1 (PMC6366028; doi:10.1186/s12916-019-1265-1)
Supplement: Supplementary file 5 — Table S5. Results of multiple logistic regression for association of stillbirth for sub-group analysis for referred deliveries with select risk factors in the Indian state of Bihar. (DOCX 12 kb) [file 12916_2019_1265_MOESM5_ESM.docx]

**Additional Table 5. Results of multiple logistic regression for association of stillbirth for sub-group analysis for referred deliveries with select risk factors in the Indian state of Bihar.**

|  |  | **Adjusted odds ratio for stillbirth**  **(95% confidence interval)*** |
| --- | --- | --- |
| **By place of delivery** | | |
| Referred delivery | Public facility |  |
| No | No | 1.00 |
| Yes | No | 3.55 (2.26-5.58) |
| No | Yes | 0.45 (0.31-0.65) |
| Yes | Yes | 3.67 (1.81-7.45) |
| **By breech presentation of the baby** | | |
| Referred delivery | Breech presentation of the baby |  |
| No | No | 1.00 |
| Yes | No | 6.45 (4.35-9.55) |
| No | Yes | 4.66 (2.72-7.97) |
| Yes | Yes | 23.08 (11.00-48.44) |

*Adjusted for place of residence and sex of the baby
